# Vasodilator Therapy: Nitrates and Nicorandil

**DOI:** 10.1007/s10557-016-6668-z

**Published:** 2016-06-16

**Authors:** Jason M. Tarkin, Juan Carlos Kaski

**Affiliations:** 1 Division of Cardiovascular Medicine, University of Cambridge, Box 110, ACCI, Addenbrooke’s Hospital, Cambridge, CB2 QQ UK; 2 Cardiovascular and Cell Sciences Research Institute, St George’s, University of London, Cranmer Terrace, Tooting, London, SW17 0RE UK

**Keywords:** Nitrates, Nicorandil, Vasodilators, Stable angina

## Abstract

Nitrates have been used to treat symptoms of chronic stable angina for over 135 years. These drugs are known to activate nitric oxide (NO)-cyclic guanosine-3′,-5′-monophasphate (cGMP) signaling pathways underlying vascular smooth muscle cell relaxation, albeit many questions relating to how nitrates work at the cellular level remain unanswered. Physiologically, the anti-angina effects of nitrates are mostly due to peripheral venous dilatation leading to reduction in preload and therefore left ventricular wall stress, and, to a lesser extent, epicardial coronary artery dilatation and lowering of systemic blood pressure. By counteracting ischemic mechanisms, short-acting nitrates offer rapid relief following an angina attack. Long-acting nitrates, used commonly for angina prophylaxis are recommended second-line, after beta-blockers and calcium channel antagonists. Nicorandil is a balanced vasodilator that acts as both NO donor and arterial K^+^_ATP_ channel opener. Nicorandil might also exhibit cardioprotective properties via mitochondrial ischemic preconditioning. While nitrates and nicorandil are effective pharmacological agents for prevention of angina symptoms, when prescribing these drugs it is important to consider that unwanted and poorly tolerated hemodynamic side-effects such as headache and orthostatic hypotension can often occur owing to systemic vasodilatation. It is also necessary to ensure that a dosing regime is followed that avoids nitrate tolerance, which not only results in loss of drug efficacy, but might also cause endothelial dysfunction and increase long-term cardiovascular risk. Here we provide an update on the pharmacological management of chronic stable angina using nitrates and nicorandil.

## Introduction

Nitroglycerine was first applied to treat stable angina in 1876, [1] and its clinical usefulness continues to this day; indeed short-acting sublingual glyceryl trinitrate (GTN) is currently recommended for all patients as the best first-line treatment for relief of acute angina symptoms [2, 3]. Long-acting nitrates, including isosorbide mononitrate (ISMN) and dinitrate (ISDN), are important second-line preventative drugs. Nitrate vasodilators are metabolized to nitric oxide (NO) within vascular smooth muscle cells, resulting in dilatation of systemic and coronary vascular beds [4]. While decades of research relating to NO signaling pathways have revealed some of the greatest discoveries in vascular biology and physiology, including seminal work by three Nobel prize laureates, [5–7] still, precise mechanisms underlying nitrate biotransformation, mode of action, and the molecular basis of nitrate tolerance remain incompletely understood [8]. Nitrates are often regarded as ‘neutral’ in regards to cardiovascular risk– however new insights suggest a combination of potentially beneficial and/or deleterious effects linked to long-term use [9].

Nicorandil is another vasodilator commonly used to treat chronic stable angina, although it is not currently available in the United States. Unlike nitrates, the actions of nicorandil result in ‘balanced’ arterial and venous dilatation, mediated via two distinct anti-angina mechanisms [10]. In addition, nicorandil is not associated with tolerance or rebound angina, and there is some (albeit inconclusive) evidence to suggest prognostic benefit due to reduction in oxidative stress during myocardial ischemic reperfusion injury [11].

Successful pharmacological management of stable angina hinges on appropriate drug selection, tailored to individual patient needs [12]. Nitrates and nicorandil are effective second-line drugs for prophylaxis of effort-induced angina, [13] as well as angina due to coronary spasm, ‘mixed’ angina, and for some patients with microvascular dysfunction [14–16]. Knowledge of specific pharmaco-therapeutic actions and tolerability profiles of these drugs can provide foresight to help optimize medical therapy and reduce frequency of unwanted side effects.

## Nitrates

Nitrate vasodilators comprise a group of organic nitrate esters with a nitrooxy (−O-NO2) moiety, which can be used as mono- or add-on therapy in combination with other anti-angina drugs [17, 18]. GTN, ISMN, and ISDN are the most frequently prescribed nitrates. Pentaerythrityl tetranitrate (PETN) is a high-potency long-acting nitrate, which is not currently recommended due to lack of clinical efficacy data [19].

### Mechanism of Action

The molecular basis of nitrate pharmacotherapy is mediated via activation of endogenous NO-cGMP signaling pathways [20]. Nitrates act as NO donors, [21, 22] possibly compensating for compromised endothelial function in patients with coronary atherosclerosis [23–25]. NO is widely accepted as the effector compound responsible for nitrate-induced vascular smooth muscle cell relaxation, [26] although several studies have reported discrepancy between nitrate vasoactivity and degree of NO release [27, 28].

While several mechanisms of nitrate bioactivation have yet to be fully elucidated, it is known that GTN and PETN are prodrugs, metabolized at therapeutic concentrations by mitochondrial aldehyde-dehydrogenase (ALDH-2) [29]. GTN is converted by ALDH-2 to its denitrated metabolite (1,2-gylceryl dinitrate) and nitrite (NO_2_); in theory, NO is then generated from either NO_2_ reduction, or as direct result of interaction between the two metabolites [30]. Presence of an ALDH-2 polymorphism resulting in Glu504Lys substitution, which eliminates ALDH2 activity, has been associated with lack of response to sublingual GTN in a Chinese patient population [31]. ISDN and ISMN bioactivation is independent of ALDH-2 activity, and in part due to cytochromal p450 metabolism in the endoplasmic reticulum, but this process is less well understood [32, 33]. After bioactivation, nitrate-generated NO activates soluble guanylate cyclase, increasing cGMP production and activation of cGMP-dependent kinases/ cyclic nucleotide-gated ion channels [34, 35]. Ultimately, this causes vasorelaxation due to reduction in intracellular free Ca^2+^ and desensitization of smooth muscle cell contractile elements to Ca^2+^ [36].

### Hemodynamic Effects

**Fig. 1:**
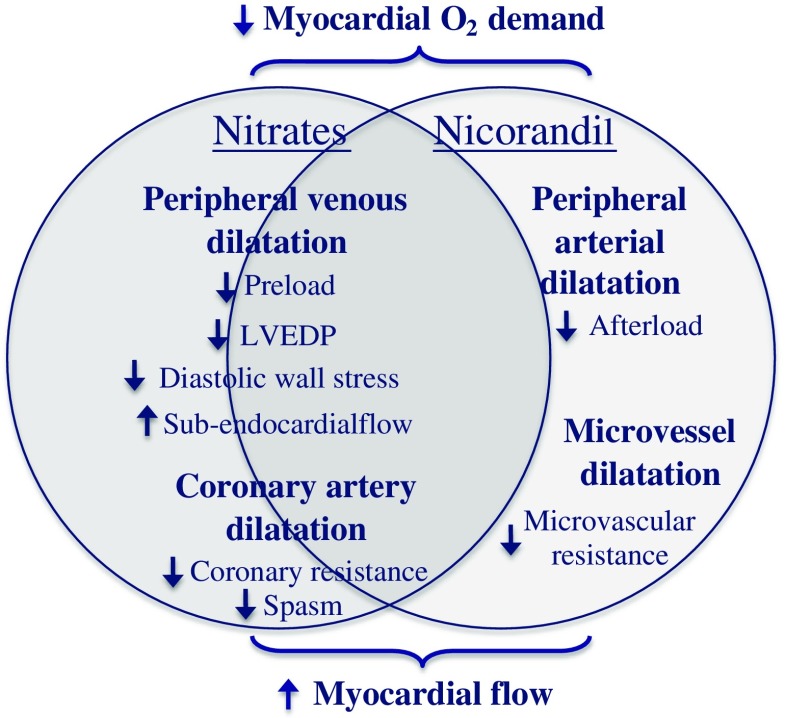
Anti-angina actions of nitrates and nicorandil

The hemodynamic effects of nitrates help to alleviate angina symptoms by reducing myocardial oxygen demand and improving myocardial blood flow (Fig. 1). At therapeutic doses, nitrates affect venous capacitance vessels predominately, but also dilate large and medium sized coronary arteries, and arterioles >100μm [37–39]. Peripheral venous dilatation results in venous pooling and thus decreased venous return, thereby lowering left ventricular end-diastolic filling pressure (preload) and volume, decreasing myocardial work and oxygen demands, and indirectly increasing sub-endocardial blood flow. At higher doses, nitrates result in arterial vasodilatation, reducing systemic vascular resistance (afterload) and blood pressure.

Myocardial blood flow is improved by dilatation of epicardial and collateral coronary arteries, particularly stenotic arterial segments prone to spasm [40–43]. GTN dilates normal coronary arteries by 18 %, and lowers coronary resistance by 38 % in arteries with severe stenosis [44]. The net hemodynamic effect of GTN on the coronary circulation replenishes myocardial blood flow to ischemic regions [45–47]. Overall, nitrates do not significantly alter cardiac output when used for chronic stable angina, and have limited effect on blood pressure at usual non-parental doses. However, as venous pooling is greatest in the upright position, this can increase the risk of orthostatic hypotension in patients taking nitrates [48].

### Non-Hemodynamic Effects and Cardiovascular Risk

Despite its long track record, consequences of chronic nitrate use remain uncertain. Nitrates have important actions beyond their role as vasorelaxants; some of these actions might be cardioprotective, whereas others have been linked to increased cardiac risk [9]. Firstly, nitrates inhibit platelet adherence and activation [49]. Increased platelet cGMP decreases fibrinogen binding to the glycoprotein IIB/IIIa receptor, which is essential for platelet aggregation [50]. The anti-platelet effects of nitrates are also mediated by NO-driven cyclooxygenase/ prostacyclin 2 activation [51, 52]. In patients receiving intravenous GTN, >50 % inhibition of platelet aggregation has been observed when tested in response to the pro-coagulant stimuli adenosine diphosphate and thrombin [53]. While the anti-platelet actions of nitrates could, in theory, be beneficial in the context of an acute coronary syndrome (ACS) this is not supported by clinical studies [54, 55]. In this context, any impact of nitrates on platelet function is probably overshadowed by the actions of potent anti-platelet drugs.

**Fig. 2:**
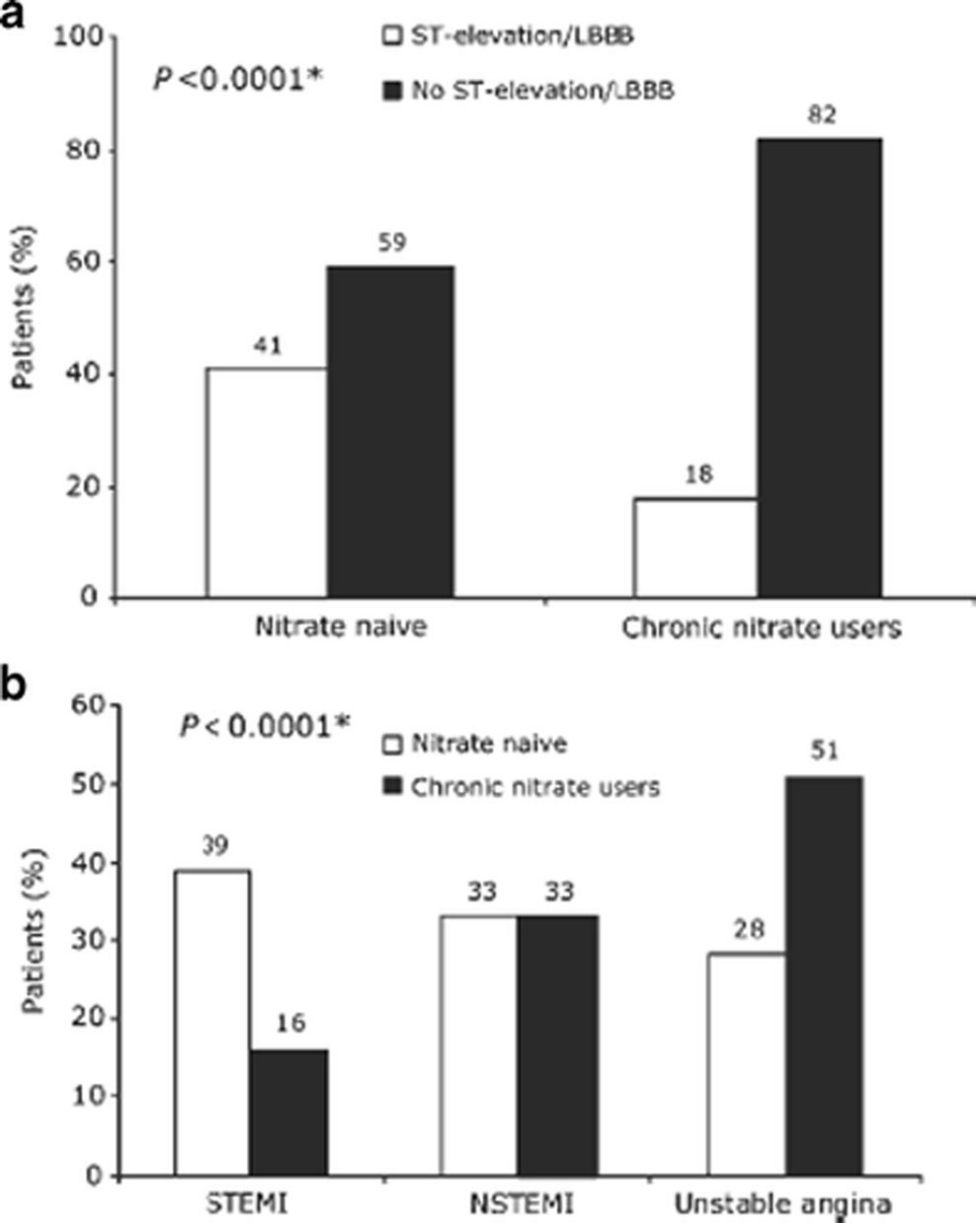
Mode of ACS presentation among patients receiving long-term nitrates vs. nitrate naïve from the Global Registry of Acute Coronary Events. *Adapted from Ambrosio* et al. *EHJ*; *2009*

Another potentially beneficial action of nitrates is activation of ischemic mitochondrial preconditioning mechanisms [56, 57]. It has been proposed that NO-induced reactive oxygen species (ROS) could have a paradoxically beneficial effect on myocardial cells through activation of protein kinase C and/or transient mitochondrial permeability transition pore (mPTP) opening during ischemic reperfusion [58–60]. In fact, nitrate-mediated ischemic preconditioning might act to alter mode of presentation in patients with ACS. Analysis of data from the Global Registry of Acute Coronary Events (GRACE), including 52,693 patients from 123 centers in 14 countries revealed that patients on long-term nitrates who present with ACS have a lower incidence of ST elevation myocardial infarction and lower cardiac enzyme release than those who are nitrate naïve (Fig. 2) [61]. While this data from GRACE confirms similar findings of an earlier study, [62] whether this link is causal remains to be determined [63].

Nitrates lose their clinical effect after 12–24 h of continuous therapy, due to drug tolerance [64]. The cause of nitrate tolerance is likely multi-factorial, and several theories have been proposed [65]. At present, the prevailing hypothesis is that tolerance results from superoxide (O_2_^−^) formation, which decreases NO bioavailability and causes uncoupling of nitric oxide synthase (NOS), resulting in impaired NO-cGMP signaling [34, 66]. Increased oxidative stress might also inhibit ALDH-2 activity [67].

Mechanisms underlying nitrate tolerance not only result in poor symptom control, but might also be potentially damaging [68–70]. Accumulation of free radicals during long-term nitrate therapy has been linked to endothelial dysfunction, [71] and increased vasoconstrictor sensitivity– providing a possible explanation for ‘rebound angina’ occurring from nitrate withdrawal [72–74]. Thus, it appears nitrate-induced ROS production might explain both protective and deleterious effects of nitrates [75]. Another potential problem is nitrate ‘pseudotolerance,’ resulting in cardiac autonomic dysfunction due to increased neurohormonal response to vasodilatation and activation of the renin-angiotensin-aldosterone system [32, 76]. Further prospective clinical studies are warranted to determine the long-term non-hemodynamic effects of nitrates, and how these factors might influence overall cardiovascular risk [3].

### Clinical Trials

Nitrates have been demonstrated in clinical trials to improve exercise tolerance, time to symptom onset, and time to ST-segment depression during exercise testing in patients with stable effort-induced angina [77]. In a meta-analysis performed in 2009 to assess usefulness of nitrates for stable angina, 51 randomized controlled, or crossover trials including a total 3595 patients were identified [78]. The authors concluded that, overall, nitrate therapy reduced the number of angina episodes by 2.45 per week, with greater effect on exercise duration and ST depression from intermittent over continuous dosing.

When taken 3–5 min before exercise testing, sublingual GTN spray abolishes or delays onset of angina and ST-segment depression with greater effect than placebo, [79] and linear dose-response [80]. Improved exercise has also been shown after 2 and 4 h of treatment with transdermal GTN, but not beyond 24 h when therapy is uninterrupted [81]. Standard release ISDN increases time to angina during exercise testing after the initial dose for up to 6–8 h, but, again, efficacy is lost with sustained three or four times daily dosing due to tolerance [82]. While three times daily buccal GTN does not cause tolerance owing to its short half-life, [83] it is important to note that partial cross-tolerance blunts the hemodynamic response to sublingual GTN when given to patients also taking sustained ISDN [84]. In a study comparing low and high doses of oral ISDN, there was no added benefit of higher dose on exercise duration [85].

The Compliance With Oral Mononitrates in Angina Pectoris Study (COMPASS) showed that patients were more likely to adhere to once daily vs. twice daily ISMN dosing regime, which overall resulted in less angina in this group [86]. In a double-blind controlled study including 313 patients with stable effort-induced angina, significantly increased exercise time was observed compared to placebo at 4 and 12 h after administration of extended release ISMN [87]. In this study, ISMN at 120 mg or 240 mg doses resulted in greater improvement in exercise tolerance than lower doses, without development of tolerance or rebound angina. Reduction in extent and severity of stress-induced ischemic defects has also been shown after treatment with this drug using Tc-99m-MIBI exercise single photon emission tomography (SPECT) [88, 89]. When evaluating ISMN dosage on quality-of-life indices, better angina control seems to result at higher doses, without significant increase in side effects [90].

### Drug Formulations, Dosage and Pharmacokinetics

Nitrates are rapidly absorbed from mucous membranes, the gastrointestinal tract and skin. Onset of action, bioavailability, and duration varies between nitrate preparations (Table 1). Plasma GTN levels are initially high after sublingual administration, but quickly disappear [91]. GTN and ISDN undergo extensive first-pass metabolism by the liver resulting in low bioavailability; short-acting GTN is therefore ineffective if swallowed. Oral ISMN is completely absorbed and, as it avoids first pass metabolism, has 100 % bioavailability, leading to a more predictable dose-response with less variation in plasma levels compared to other nitrates [92].

**Table 1:** Prescribing nitrates and nicorandil

Drug	First-pass effect	Pro-drug	Usual dose	Frequency	Max dose	Onset	Duration
Nitrates							
Glycerol-trinitrate (GTN)							
Sublingual tab	No	Yes	0.3–0.6 mg	As needed (every 5 mins)	1.5 mg	1–3 min	10–30 min
Sublingual spray	No	Yes	0.4 mg/dose	As needed (every 5 mins)	1.5 mg	1–3 min	10–30 min
Patch	No	Yes	0.1–0.8 mg/h	1 daily (12 h on/ 12 h off)	1 patch/ day	30 mins	8–14 h
Isosorbide dinitrate (ISDN)							
Sublingual tab	No	Yes	2.5 mg	Repeated as needed	15 mg	3–4 min	1 h
Chewable tab	No	Yes	5 mg	Repeated as needed	15 mg	3–4 min	1 h
Standard release	Yes	Yes	30–120 mg	2–3 daily (last dose 6pm)	240 mg/ day	15–30 min	3–6 h
Extended relsease	Yes	Yes	20–40 mg	2 daily (8am and 3pm)	80 mg/ day	30–60 min	12–14h
Isosorbide mononitrate (ISMN)							
Standard release	No	Yes	10–40 mg	2 daily (8am and 3pm)	120 mg/ day	30–60 min	6–8 h
Extended release	No	Yes	60–240 mg	Once daily	240 mg/ day	30–60 min	12–14h
K^+^ATP agonist							
Nicorandil	No	Yes	10–20 mg	2 daily (8am and 3pm)	60 mg/ day	30–60 min	12 h

GTN is typically given for acute angina relief as a sublingual tablet (0.3–0.6mg) or spray (0.4mg/dose). Its onset of action is 1–3 min, and duration of action 10–30 min. Due to the risk of orthostatic hypotension, patients are advised to take GTN while seated. Doses can be repeated at 5 min intervals until the pain is resolved, but prompt medical attention should be sought when chest pain is severe or persists >15 min. Buccal GTN and ISDN can also be given for acute angina relief as sublingual tablet (2.5 - 15mg), chewable tablet (5mg), or spray (1.25mg/dose). Buccal GTN and shorter-acting ISDN preparations take several minutes longer than sublingual GTN to work, but last for >1 h. When taken orally, the onset of action of standard release ISDN is 15–30 min and duration of action 3–6 h. For standard release ISMN this is 30–60 min and 6–8 h respectively [93, 94].

Nitrate tolerance can be avoided with eccentric dosing regimes and use of extended release formulations, plus a daily nitrate-free interval of at least 8–10 h [87, 95]. There is also some evidence that folic acid, L-arginine, Vitamin C, and other anti-oxidants can help to prevent nitrate tolerance and endothelial dysfunction [71, 96, 97]. However, tolerance develops with all currently available long-acting nitrates and none can provide sustained 24 h prophylaxis [98]. A typical starting dose of extended release ISMN is 30 mg or 60 mg once daily, which can be up-titrated to 120 or 240 mg if required. A single dose of extended release ISMN provides cover for up to 12–14 h. When transdermal GTN is used, tolerance is avoided by interrupting patches with regular nitrate free (patch-off) breaks [99, 100]. Although nighttime patch removal can circumvent nitrate tolerance, this approach does not provide prophylaxis against nocturnal angina; and might actually worsen angina during this period due to the rebound effect of nitrate withdrawal, and ‘zero-hour’ effect resulting in worsened exercise tolerance in the morning before patch application [65]. Rebound angina is not seen with long-acting oral nitrates.

### Side Effects and Tolerability

Headache is the most common side effect of nitrates, which prevents its use 10 % of patients [101]. Headaches occurring within the first hour of nitrate administration are usually due to vasodilation, which can be avoided by use of a low starting dose with slow up-titration [102]. Simple headaches typically disappear within 1–2 weeks of treatment, and co-administration with aspirin given for secondary prevention can help to reduce this symptom. Nitrates can, however, also trigger migraine and other types of headache [103]. Other common side effects of nitrates are: light-headedness, flushing, and postural hypotension with risk of syncope (Table 2).

**Table 2:** Side effects of nitrates and nicorandil

Drug	Side-effects	Contraindications
Nitrates		
	>10: headache	Hypotension
		Cardiogenic shock
	0.1–10 %: dizziness, flushing, nausea, vomiting, light-headedness, orthostatic hypotension, syncope, contact dermatitis (patch)	Hypertrophic cardio myopathy severe Aortic stenosis PDE-5 inhibitors closed angle glaucoma
	Rare: methaemoglobinaemia	
Nicorandil		
	Common: as per nitrates	As per nitrates
	Rare: skin and gastric ulceration	

### Drug Safety and Cautions

The safety of nitrates has been demonstrated during its many years of clinical use. Risk of orthostatic hypotension is greater in the elderly due to age-related autonomic dysfunction. If syncope occurs, nitrates should be discontinued. Nitrates are contra-indicated in patients with hypertrophic cardiomyopathy, and used with caution in aortic stenosis due to risk of worsening outflow tract gradient. Other absolute contraindications to nitrates are co-administration with phosphodiesterase-5 (PDE-5) inhibitors (e.g. sildenafil) due to the risk of profound hypotension, and closed angle glaucoma. Methemoglobinemia is a rare adverse effect, which has been reported with large nitrate doses. Safety of nitrates in pregnancy and breastfeeding has not been evaluated, and therefore should be avoided when possible.

## Nicorandil

Nicorandil (*N*-[2-(Nitro-oxy) ethyl]-3-pyridine carboxamide) is a balanced vasodilator, which affects both venous and arterial beds. Its chemical structure consists of a nicotinamide derivative combined with nitrate moiety. Overall, nicorandil is similarly effective for angina prophylaxis to long-acting nitrates and other conventional anti-anginal drugs, however it does not cause tolerance and might offer added prognostic benefit [104, 105].

### Mechanism of Action

Nicorandil exerts two distinct anti-angina mechanisms, acting as both: (1) NO donor, and (2) K^+^_ATP_ channel opener [106]. The nitrate-like action of nicorandil accounts for the majority of its clinical efficacy at therapeutic concentrations. [107]. Bioactivation of nicorandil involves denitration via the nicotinamide/ nicotinic acid pathway [108]. Similar to nitrates, NO acts via cGMP signaling pathways within vascular smooth muscle cells causing peripheral and coronary vasodilatation [10]. In addition, its action on K^+^_ATP_ channels results in vascular smooth cell hyperpolarization and closure of L-type voltage gated calcium channels, [109] which acts to dilate both coronary microvessels and peripheral resistance arteries [110, 111].

### Hemodynamic Effects

The hemodynamic effects of nicorandil result in balanced offloading of the ventricles through reduction in preload and afterload, and improved coronary flow due to lowered coronary arterial resistance [112, 113]. Nicorandil dilates coronary arteries by 10–20 % in patients with coronary atherosclerosis, mostly because of its nitrate-like effect [114]. Coronary dilatation induced by nicorandil occurs in both normal and diseased vessel segments, [115] with potentially greater effect on stenotic areas than nitrates [116]. In one study, 40 mg of nicorandil administered via oral or sublingual routes led to significant reduction in left ventricular end-diastolic pressure after 15 mins, and total peripheral resistance was reduced by 19 % [117]. Similar to nitrates, a mild dose-dependent baroceptor reflex tachycardia can also occur. Nicorandil does not directly affect cardiac conduction or contractility.

### Non-Hemodynamic Effects and Cardiovascular Risk

The actions of nicorandil on K^+^_ATP_ channels are thought to confer cardioprotection through activation of pathways linked to ischemic preconditioning [118, 119]. Nicorandil also appears to have a protective effect on endothelial function, [120–123] and might help to stabilize coronary plaque [124]. Several theories have been proposed to explain potential cardioprotective properties of nicorandil. One hypothesis is that K_ATP_ channel opening mimics actions of endogenous adenosine release, thereby shortening myocardial cell action potentials, and reducing Ca^2+^ overload and cellular energy demands [125, 126]. However, increasing evidence highlights the role of mitochondrial K_ATP_ channels in mediating ischemic preconditioning due to nicorandil, [127, 128] and its potential to prevent oxidative damage through inhibition of mPTP activation during ischemic reperfusion injury [11, 129]. Nicorandil might also prevent myocardial ischemic reperfusion injury through suppression of endoplasmic reticulum stress-induced apoptotic cell death through the PI3K/Akt pathway [130].

Data from several randomized controlled trials suggest that treatment with nicorandil might improve long-term clinical outcomes. This potential benefit was first suggested by the Impact of Nicorandil in Angina (IONA) study [131]. In IONA, 5126 patients with stable angina were randomized to receive 20 mg of nicorandil or placebo, with mean follow up of 1.6 ± 0.5 yrs. Results of IONA showed a significant reduction in the composite end-point of death due to coronary heart disease, non-fatal myocardial infarction or unplanned hospital admission with chest pain in the treatment group 13.1 % vs. placebo 15.5 %, HR 0.83, *p* = 0.014 (Fig. 2). This observation occurred independently of any impact from subgroup status, [132] however IONA was underpowered to show statistical significance for the secondary outcome of coronary heart disease mortality or non-fatal myocardial infarction, and the individual components of the composite end point did not differ significantly between the two study groups [133]. Further prognostic evidence for nicorandil comes from the Japanese Coronary Artery Disease (JCAD) study [134]. JCAD was a multi-centre prospective observational study, which compared outcomes of 2558 patients with ≥75 % epicardial artery stenosis treated with nicorandil vs. matched controls. The follow up period was 2.7 yrs. Results of JCAD showed a 35 % reduction in all cause mortality (HR 0.65, *p* = 0.0008) and 56% reduction in cardiac death (HR 0.44, *p* < 0.0001) in the treatment group. There is also some evidence that nicorandil reduces risk of non-sustained ventricular and supraventricular arrhythmia when used in patients with unstable angina [135]. However, whether or not nicorandil provides any prognostic benefit in addition to its anti-angina effects has yet to be determined conclusively, and therefore the current indication for its use in stable angina is for symptomatic relief.

## Clinical Efficacy

The clinical efficacy of nicorandil for treatment of effort-induced stable angina has been evaluated by a number of clinical trials. Open label, placebo-controlled and comparative studies have demonstrated reduction in frequency of angina episodes and improvement of exercise tolerance following treatment with nicorandil [136–142]. In one placebo-controlled study, time to angina during exercise testing was increased by 38 % at 2 h and 23 % at 12 h after two-weeks of nicorandil [143]. A meta-analysis of 20 prospective controlled trials showed that, overall, nicorandil is equally effective to standard angina treatment [144].

Positive efficacy data exist for nicorandil when compared to long-acting nitrates, [145, 146] calcium channel blockers, [147, 148] and beta-blockers [149–151]. The Study of Nicorandil in Angina Pectoris in the Elderly (SNAPE), which included 194 patients, reported similar improvement in time to angina and time to ST depression during symptom-limited bicycle exercise testing after 4 weeks of nicorandil vs. ISMN [152]. Comparison of the Anti-ischemic and Anti-anginal Effects of Nicorandil and Amlodipine in Patients with Symptomatic Stable Angina Pectoris (SWAN) study was a multi-centre, double-blind, randomized study of 121 patients from 25 centers in Austria and Switzerland [153]. SWAN showed comparable performance and tolerability for these two angina drugs.

### Dosage and Pharmacokinetics

Nicorandil is rapidly and almost completely absorbed via the gastrointestinal tract, reaching maximal plasma concentration after 30–60 min, and steady-state levels following 4–5 days of standard therapy. Gastrointestinal absorption is delayed, but not decreased by food. Its half-life is roughly 52 mins. Nicorandil does not undergo first-pass metabolism, and displays a linear dose-to-plasma concentration relationship at doses of 5 - 40mg. Its oral bioavailablity is >75 %, with <20 % of the drug excreted in the urine. Nicorandil circulates largely unbound to albumin or other plasma proteins. Its anti-angina effects last approximately 12 h, necessitating twice-daily dosage. Pharmacokinetic properties are not significantly effected by age, chronic liver and/or renal disease [108, 154].

A usual starting dose of nicorandil is 10 mg twice daily, or 5 mg for patients susceptible to headache. The lowest effective dose is recommended to avoid potential side effects, especially in the elderly. The therapeutic dose is typically 10 - 20 mg twice daily, and maximum dose 30 mg twice daily. Unlike nitrates, tolerance to nicorandil does not tend to occur, probably because of its dual mode of action [155, 156]. However, an attenuated response during exercise testing was reported in one study after 2 weeks of sustained therapy. Nicorandil does not cause rebound angina [157].

### Side Effects and Tolerability

Nicorandil is well tolerated by most patients. Less than 10 % of patients report side-effects after 30 days of treatment, and roughly 70 % remain on nicorandil at 1 year [158]. Similar to nitrates, headache is the most common side effect to nicorandil, occurring in roughly one third of patients. Other relatively common side effects are: dizziness, flushing, malaise and gastro-intestinal upset. Rarely, nicorandil can cause gastrointestinal, skin, mucosal, or eye ulceration [159, 160]. Nicorandil should be stopped immediately if ulceration occurs.

### Drug Safety and Cautions

Similar to nitrates, nicorandil is contraindicated in the setting of compromised blood pressure and must not be used in conjunction with PDE-5 inhibitors due to the risk of severe hypotension resulting from this dangerous drug combination. Due to risk of gastro-intestinal ulceration, caution is advised when prescribing nicorandil for patients also taking corticosteroids. Although the overall safety of nicorandil has been demonstrated by numerous clinical trials, including the Prescription Event Monitoring (PEM) study, [161] recent review by the European drug regulatory agencies have lead the manufacturer to highlight several additional contraindications and warnings. The manufacturer now states that gastro-intestinal ulcers can progress to perforation, hemorrhage, fistula or abscess – and that patients with diverticular disease might be at higher risk of these severe complications. Ulcers caused by nicorandil do not respond to conventional ulcer treatment, including surgery, and therefore if ulcers occur in any part of the body nicorandil must be stopped. Actions of nicorandil on K^+^_ATP_ channels are antagonized by some sulphonyureas, including glibenclamide and glimepiride [162]. The effects of nicorandil during pregnancy, breast-feeding and on fertility have not been studied in humans and therefore this agent should be avoided in this context.

## Coronary Spasm and Microvascular Angina

**Fig. 3:**
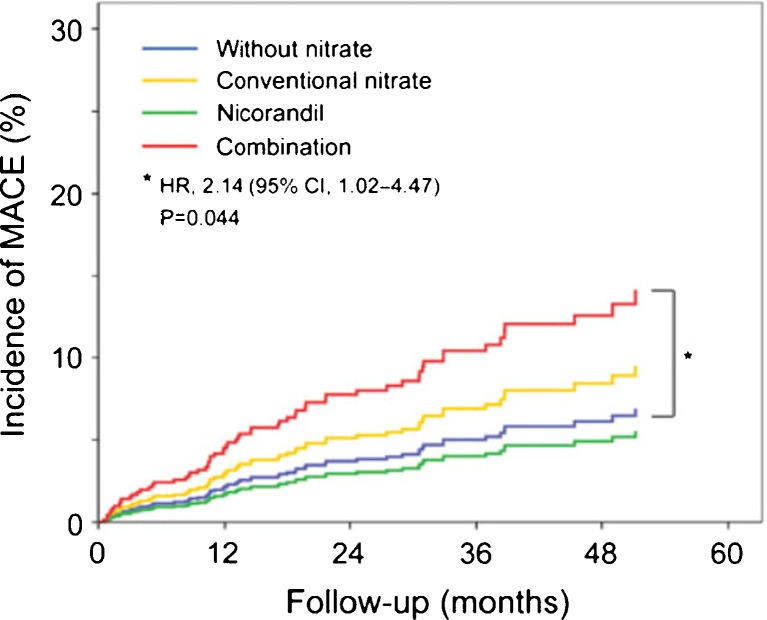
Cumulative incidence of major adverse cardiac events (MACE) occurring in patients treated with nitrates and/ or nicorandil for vasospatic angina in the multi-centre registry study of the Japanese Coronary spasm association. *Adapted from Takahashi* et al. *EHJ*; *2014*

Although calcium channel blockers are the drug of choice for the treatment of angina resulting from coronary artery spasm, vasospastic angina can also be successfully treated with nitrates and nicorandil [163, 164]. However, recent data from the Japanese coronary spasm association registry indicates a potentially higher incidence of major adverse cardiac events (MACE) when these two drugs are used together for treatment of chronic vasospastic angina (Fig. 3) [165]. Nitrates have limited use in patients with microvascular angina owing to its relatively small vasodilatory effect on small resistance vessels [166]. In clinical practice, however, they are useful in roughly 50 % of patients [167, 168]. Nicorandil has a more pronounced effect on the coronary microcirculation than nitrates, and therefore might be better suited for patients with microvascular angina, including those with microvascular spasm [169–172].

## Conclusion

Long-acting nitrates and nicorandil are effective drugs for treatment of stable effort-induced angina, which are recommended ‘second-line’ according to current European guidelines. These pharmacological agents are particularly useful for patients who are able to tolerate short-acting GTN without side effects. When compared to other second-line anti-angina drugs (e.g. ranolazine and ivabradine), nitrates and nicorandil are on the whole similarly effective to these agents, and choice is guided by individual factors such as co-morbidities, contra-indications, availability and patient preference [133]. A major limitation of nitrate therapy is drug tolerance. While tolerance can be avoided by dosing regimes that incorporate a nitrate-free interval, the risk of nocturnal angina and rebound angina remains. Further studies are warranted to determine the long-term impact of nitrates on cardiovascular outcomes. One advantage of nicorandil over long-acting nitrates is that tolerance does not occur, but there are other potential adverse effects of this drug to be considered, including, rarely, skin or gastro-intestinal ulceration. There is also some evidence from large prospective clinical trials to suggest that nicorandil might improve long-term clinical outcomes. However, further research is needed to understand the potential cardioprotective mechanisms of nicorandil and to assess its impact on long-term survival. While each drug has potential advantages and limitations, long-acting nitrates and nicorandil are important pharmacological agents for management of chronic stable angina triggered by obstructive atherosclerotic coronary artery disease, as well as microvascular angina and epicardial coronary artery spasm.
