# Erratum to: Vasodilator Therapy: Nitrates and Nicorandil

**DOI:** 10.1007/s10557-017-6744-z

**Published:** 2017-08-14

**Authors:** Jason M. Tarkin, Juan Carlos Kaski

**Affiliations:** 1 Division of Cardiovascular Medicine, University of Cambridge, Box 110, ACCI, Addenbrooke’s Hospital, Cambridge, CB2 QQ UK; 2 Cardiovascular and Cell Sciences Research Institute, St George’s, University of London, Cranmer Terrace, Tooting, London, SW17 0RE UK

**Erratum to: Cardiovasc Drugs Ther** (**2016) 30:367–378**

**https://doi.org/10.1007/s10557-016-6668-z**

The article entitled Vasodilator Therapy: Nitrates and Nicorandil, written by Jason M. Tarkin and Juan Carlos Kaski, was originally published Online First without open access. After publication in volume 30, issue 4, pages 367–378, the author decided to opt for Open Choice and to make the article an open access publication. Therefore, the copyright of the article has been changed to © The Author(s) [2016] and the article is forthwith distributed under the terms of the Creative Commons Attribution 4.0 International License (http://creativecommons.org/licenses/by/4.0/), which permits use, duplication, adaptation, distribution and reproduction in any medium or format, aslong as you give appropriate credit to the original author(s) and the source, provide a link to the Creative Commons license, and indicate if changes were made.

